# A mouse model of the 15q13.3 microdeletion syndrome shows prefrontal neurophysiological dysfunctions and attentional impairment

**DOI:** 10.1007/s00213-016-4265-2

**Published:** 2016-03-17

**Authors:** Simon R. O. Nilsson, Pau Celada, Kim Fejgin, Jonas Thelin, Jacob Nielsen, Noemí Santana, Christopher J. Heath, Peter H. Larsen, Vibeke Nielsen, Brianne A. Kent, Lisa M. Saksida, Tine B. Stensbøl, Trevor W. Robbins, Jesper F. Bastlund, Timothy J. Bussey, Francesc Artigas, Michael Didriksen

**Affiliations:** Department of Psychology, University of Cambridge, Cambridge, CB2 3EB UK; MRC and Wellcome Trust Behavioural and Clinical Neuroscience Institute, University of Cambridge, Cambridge, CB2 3EB UK; Department of Psychology, State University of New York at Binghamton, Binghamton, NY 13902-6000 USA; Institut d’Investigacions Biomèdiques de Barcelona, CSIC-IDIBAPS, Barcelona, Spain; Centro de Investigación Biomédica en Red de Salud Mental (CIBERSAM), Barcelona, Spain; H. Lundbeck A/S, Synaptic Transmission, Neuroscience Research DK, Ottiliavej 9, Valby, 2500 Denmark; Neuronano Research Center, Lund University, 223 81 Lund, Sweden; Department of Life, Health and Chemical Sciences, The Open University, Walton Hall, Milton Keynes, MK7 6AA UK

**Keywords:** Copy number variation, 15q13.3, Animal model, *Chrna7*, Prefrontal cortex, Neurophysiology, Cognition

## Abstract

**Rationale:**

A microdeletion at locus 15q13.3 is associated with high incidence rates of psychopathology, including schizophrenia. A mouse model of the 15q13.3 microdeletion syndrome has been generated (Df[h15q13]/+) with translational utility for modelling schizophrenia-like pathology. Among other deficits, schizophrenia is characterised by dysfunctions in prefrontal cortical (PFC) inhibitory circuitry and attention.

**Objectives:**

The objective of this study is to assess PFC-dependent functioning in the Df(h15q13)/+ mouse using electrophysiological, pharmacological, and behavioural assays.

**Method:**

Experiments 1–2 investigated baseline firing and auditory-evoked responses of PFC interneurons and pyramidal neurons. Experiment 3 measured pyramidal firing in response to intra-PFC GABA_A_ receptor antagonism. Experiments 4–6 assessed PFC-dependent attentional functioning through the touchscreen 5-choice serial reaction time task (5-CSRTT). Experiments 7–12 assessed reversal learning, paired-associate learning, extinction learning, progressive ratio, trial-unique non-match to sample, and object recognition.

**Results:**

In experiments 1–3, the Df(h15q13)/+ mouse showed reduced baseline firing rate of fast-spiking interneurons and in the ability of the GABA_A_ receptor antagonist gabazine to increase the firing rate of pyramidal neurons. In assays of auditory-evoked responses, PFC interneurons in the Df(h15q13)/+ mouse had reduced detection amplitudes and increased detection latencies, while pyramidal neurons showed increased detection latencies. In experiments 4–6, the Df(h15q13)/+ mouse showed a stimulus duration-dependent decrease in percent accuracy in the 5-CSRTT. The impairment was insensitive to treatment with the partial α_7_nAChR agonist EVP-6124. The Df(h15q13)/+ mouse showed no cognitive impairments in experiments 7–12.

**Conclusion:**

The Df(h15q13)/+ mouse has multiple dysfunctions converging on disrupted PFC processing as measured by several independent assays of inhibitory transmission and attentional function.

**Electronic supplementary material:**

The online version of this article (doi:10.1007/s00213-016-4265-2) contains supplementary material, which is available to authorized users.

## Introduction

The 15q13.3 microdeletion syndrome (15q13.3DS) is caused by a rare (∼1:30000 births) (LePichon et al. 2010) copy number variant (CNV) with hemizygosity of at least seven genes on the long arm of chromosome 15. The microdeletion is associated with variable cognitive impairment and psychiatric or neurological disorders (Miller et al. 2009), including autism (Pagnamenta et al. 2009; Ben-Shachar et al. 2009), attention deficit hyperactivity disorder (Miller et al. 2009), obsessive compulsive disorder (Melchior et al. 2013), and epilepsy (Helbig et al. 2009). 15q13.3DS is also associated with a ∼10-fold increase in the risk of schizophrenia (International Schizophrenia Consortium 2008; Stefansson et al. 2014), making it one of the strongest known genetic risk factors for the disorder.

Among other alterations, the pathophysiology of schizophrenia is underpinned by imbalances within local prefrontal cortical (PFC) circuitry, typically observed as reductions in markers for cortical inhibitory signalling efficacy in histological (Lewis et al. 2005) and neurophysiological assays (Daskalakis et al. 2007; Javitt et al. 2008). Moreover, schizophrenia is characterised by core prefrontal-dependent “executive” cognitive impairments (Robbins 1990), perhaps most notably within the domain of attention (Carter and Barch 2007). Deficits in executive functioning have also been linked to dysregulated cortical recruitment and asynchronous activity partly produced by disrupted inhibitory activity during cognitive demand (Bickel and Javitt 2009; Nakazawa et al. 2012). Indeed, schizophrenia-related dysfunctions observed in measures of event-related neuronal responses are associated with attentional impairment (Nieman et al. 2002). The critical 15q13.3 segment also encompasses the *CHRNA7* gene, which is involved in cortical inhibitory transmission (Adams et al. 2012; Lin et al. 2014), regulates schizophrenia-relevant neurophysiological markers (Hajos et al. 2005), and is associated with attentional dysfunction (Adler et al. 1998; Young et al. 2004). The cognitive phenotypes associated with 15q13.3DS remain imprecisely defined and unspecific deficits typically ranging from moderate to mild intellectual disability have been described (Lowther et al. 2014; Gillentine and Schaaf 2015). The 15q13.3DS has also been associated with attentional impairments that can be independent of general intellectual functioning (Miller et al. 2009).

Preclinical lesion and local microinfusion studies have shown attentional functioning to be contingent on the activity in the medial prefrontal cortex (mPFC), including the prelimbic cortex (PrL) (Muir et al. 1996; Chudasama and Muir 2001; Passetti et al. 2002) and GABAergic activity in this area (Paine et al. 2011; Pehrson et al. 2013; Pezze et al. 2014). Recordings in animals during cognitive testing further indicate that inhibitory PrL-activity correlates with attentional performance in the 5-choice serial reaction time task (5-CSRTT) (Totah et al. 2009). We have previously generated a mouse model of the 15q13.3DS (Df[h15q13]/+) which shows 50 % reduction in messenger RNA (mRNA) expression of the genes in the region and several 15q13.3DS- and schizophrenia-like phenotypes, including decreases in the amplitude of cortical auditory-evoked potentials and aberrant responding to GABA_A_ receptor (GABA_A_R) antagonism in seizure assays (Fejgin et al. 2014).

As part of the NEWMEDS initiative (Innovative Medicines Initiative Grant Agreement No. 115008), the current study tested the translational utility of the Df(h15q13)/+ mouse for schizophrenia-relevant cortical-dependent dysfunctions using multiple parallel experimental approaches. These approaches included measures of (i) pre-attentative processing through auditory-evoked neural responses, (ii) higher order executive attention through the touchscreen 5-CSRTT, and (iii) mechanistic processes through concurrent intra-PFC GABAergic pharmacology and measures of pyramidal neuron spontaneous discharge rates.

## Methods and materials

### Animals

The Df(h15q13)/+ mouse was generated by Taconic Artemis (Köln, Germany) as previously described (Fejgin et al. 2014). The Df(h15q13)/+ mice were of a c57BL/6NTac background. All experiments used male mice. Animals were housed under a 12-h light/dark cycle with stable temperature and humidity conditions with ad libitum access to food and water. Experiments 1–2 investigated baseline and auditory-evoked neural responses of putative pyramidal cells and interneurons and used 14 mice (age 10–14 weeks; wild type (WT) *N* = 7, Df(h15q13)/+ *N* = 7). Experiment 3 analysed pyramidal neuron responses to GABA_A_R antagonism and used 19 mice (age 10–14 weeks; WT *N* = 10, Df(h15q13)/+ *N* = 9). Experiments 4–12 assessed cognitive functions and used three cohorts of animals (each cohort: WT *N* = 16, Df(h15q13)/+ *N* = 16; age at start of testing: 10 weeks). Prior to cognitive testing, animals were food restricted and maintained at about 85 % of their free-feeding weight. Cohort 1 was tested on the 5-CSRTT (experiments 4–6) and novel object recognition (experiment 12). Cohort 2 was tested on visual discrimination and reversal learning (experiment 7), paired-associate learning (PAL) (experiment 8), trial-unique non-match to sample (TUNL) (experiments 11), and extinction learning (experiment 10). Cohort 3 was tested on progressive ratio (experiment 9). The experiments followed the European Union regulation (directive 2010/63 of 22 September 2010) and UK Animals (Scientific Procedures) Act 1986 and were approved by the Barcelona School of Medicine Institutional Animal Care and Use Committee and the Danish National Committee for Ethics in Animal Experimentation.

### Experiments 1–2: cortical auditory-evoked neuronal firing

#### Surgical procedures

Mice were anesthetised with Sevoflurane (Abbott Scandinavia AB, Solna, Sweden), and body temperature was maintained at 37 °C by an isothermal heating pad. A 16-channel array electrode (Innovative Neurophysiology, Durham, NC) was placed in the PrL at stereotaxic coordinates (from bregma and duramater) AP: +1.9 mm, L: +0.2 mm, and DV: -1.5 mm according to the mouse brain atlas (Paxinos and Franklin 2008) and secured through a set of skull-mounted anchor screws (over the cerebellum and contralateral to the prefrontal cortex electrode) and dental cement (3M relyX unicem self-adhesive universal resin cement). A small hole for ground wires was drilled adjacent to the cerebellum anchor screw. Mice were treated with a prophylactic antibiotic (5 mg/kg, s.c., Baytril vet®, 50 mg/ml Enrofloxacin, Bayer, Germany) and peripheral analgesic (1.5 mg/kg, s.c., Rimadyl vet®, 50 mg/ml Carprofen, Pfizer, USA) pre-surgery and for 5 days post-surgery. Mice were allowed ≥1 week of post-surgical recovery.

#### Signal acquisition

Activity of a large number of PrL neurons was recorded in freely behaving animals. Single- and multi-unit activities were recorded using a multichannel recording system (Plexon Inc. Dallas, TX). Continuously recorded data was band pass-filtered in order to avoid low frequency activity and visualise the action potentials. Frequencies below 300 Hz were filtered to delete the slow components of the raw data, and the upper cut-off frequency of 9000 Hz was applied to diminish the noisy appearance of the action potential shape (Quian Quiroga 2009). After filtering, action potentials were easily visualised on top of background noise activity and could be detected by using an amplitude threshold. Storage threshold for spiking events was three standard deviations of the peak height histogram of filtered signal. Artifact waveforms were removed, and the spike waveform minima were aligned using the Offline Sorter software (v3.2; Plexon, Dallas, TX).

#### Spike sorting and classification

Action potentials were sorted into unit clusters using the Offline Sorter software. Waveform features used for separating the units were the first three principal components of the sampled waveforms for a given dataset. A cluster was classified as a single unit when <0.3 % of the spikes occurred within a 2-ms refractory period. To group units in putative cell types, we extracted two features from the average waveform: valley full width at half maximum and peak-to-valley time. The waveform features were plotted and used to classify the units as putative pyramidal cells or putative fast-spiking interneurons (Barthó et al. 2004) (see Supplementary Fig. 1a). Waveforms with a valley full width at half maximum duration <0.35 ms and valley to peak time <0.2 ms were considered interneurons, while waveforms with a half amplitude duration >0.37 ms and valley to peak time >0.15 ms were considered pyramidal cells. Waveforms not falling into one of the two groups were considered as intermediates and were not included for further analysis.

#### Auditory gating paradigm

Mice were tested in the dark phase and were habituated to the recording chamber for 40 min prior to the beginning of the session. Motor activity was manually monitored via an infrared camera, and recordings were made during resting state where periods with locomotor activity were excluded from analysis. Paired white noise auditory stimuli (white noise, 82 dB, 10 ms duration with 1 ms rise and fall, inter-stimulus interval 500 ms) were presented with an inter-pair interval of 10 s, and simultaneous recordings of single-unit activity were made for 10 min. A transistor-transistor logic (TTL) pulse 50 ms prior to the sound was used as event mark and saved together with single-unit data. Startle responses were not observed during the recording sessions. Peri-stimulus time histograms (PSTH) were constructed for each spike train with a bin width of 1 ms. The average PSTH across units were calculated after baseline subtraction (baseline interval: −1.4 to −0.4 s relative to first pulse) of each unit PSTH. The response amplitude was measured in a 30-ms bin starting 10 ms after the stimulus onset. The latency was set to the first 1 ms bin that was significantly higher than the baseline firing rate.

### Experiment 3: intra-mPFC GABA_A_R antagonism and pyramidal discharge rate

#### Surgical procedures

Mice were anesthetised by acute dose of chloral hydrate (400 mg/kg i.p) and maintenance dose of ∼1 mg/kg/min i.p using a perfusion pump. A heating pad maintained body temperature at 37 °C. Single-unit recordings were performed using glass micropipettes pulled from 2-mm capillary glass (World Precision Instruments, Sarasota, FL) and a Narishige PE-2 pipette puller (Tokyo, Japan). Recorded signals were amplified (×10; Neurodata IR283 amplifier, Cygnus Technology Inc., Delaware Water Gap, PA), post-amplified and filtered (×100; band pass filter: 30 Hz–10 kHz) (Cibertec amplifier/filter, Madrid, Spain) and analysed using a DAT 1401plus interface system (Spike2, Cambridge Electronic Design, Cambridge, UK).

#### Intra-mPFC gabazine

Spontaneous discharge rates of putative pyramidal neurons in Df(h15q13)/+ and WT mice were analysed during control conditions and following local GABA_A_R antagonism as described in rat PFC (Lladó-Pelfort et al. 2012). Electrodes (impedances: 6–12 MΩ) containing saline 2 M or gabazine (20 mM, dissolved in 0.2 M NaCl; SR95531, Sigma-Aldrich, St Louis, MO) were placed at stereotaxic coordinates (from bregma) AP: +2.1 mm, L: −0.2–0.4 mm. Pyramidal neuron discharge was examined in the same mice in standard (NaCl) or gabazine conditions by performing track descents in both conditions. Electrode descents were made at DV: −1–2.5 mm (from duramater) according to the mouse brain atlas (Paxinos and Franklin 2008). Pyramidal neurons were identified by their electrophysiological characteristics: (i) duration of the depolarisation phase of the action potential (average of 10 spikes) and (ii) discharge rate (Lladó-Pelfort et al. 2012) (see Supplementary Fig. 1b). Gabazine was chosen due to its superior selectivity relative to the classical GABA_A_R antagonist bicuculline (Debarbieux et al. 1998; Stocker et al. 1999). Electrode tips (5–7-μm diameter) were broken to a final resistance of 9–15 MΩ. Gabazine reached recorded neurons through passive diffusion from the electrode as previously shown for bicuculline (Steward et al. 1990; Tepper et al. 1995). To obtain a reliable measure of spontaneous discharge rate, a 5-min recording was made for each putative pyramidal neuron after which the electrode was descended again.

### Experiments 4–6: the 5-choice serial reaction time task

#### Apparatus

The 5-CSRTT experiments used 32 operant touchscreen chambers (Campden Instruments, Loughborough, UK) extensively described elsewhere (Horner et al. 2013; Mar et al. 2013).

#### 5-choice serial reaction time task (5-CSRTT)

The 5-CSRTT is a rodent analogue of the human continuous performance task (CPT) measuring attention in which schizophrenic patients show impairments (Kahn et al. 2012). The 5-CSRTT is sensitive to mPFC lesions (Muir et al. 1996; Chudasama and Muir 2001; Passetti et al. 2002) and perturbations to mPFC inhibitory signalling in the rodent (Paine et al. 2011; Pehrson et al. 2013; Pezze et al. 2014) and impulsive and attentional impairments in mouse models of neuropsychiatric disorders (Young et al. 2004; Hoyle et al. 2006; Romberg et al. 2011). The task is described in extensive detail elsewhere (Mar et al. 2013). Briefly, animals were initially trained to respond to a white-square stimulus using a 2-s stimulus duration (SD), 40-trial session length, and 5-s delay. Once the animals had acquired the criterion of ≥80 % accuracy and ≤20 % for two consecutive sessions, they were tested on a series of probe tests. Each probe test lasted for two consecutive sessions, and the presented data represents the means for these 2 days (Romberg et al. 2011). These probe tests were always presented in order of increasing difficulty (i.e., SD probes were presented in order of decreasing durations, and delay probes were presented in order of increasing delays). During probe tests the animals were tested for 7 days a week. Between each probe test, the animals had to reacquire the criteria of ≥80 % accuracy and ≤20 % omissions for two consecutive sessions on the baseline task parameters of 2-s SD and 5-s delay. These baseline sessions were done in order to reduce potential proactive interference from previous probe tests (Romberg et al. 2011; Mar et al. 2013).

In experiment 4, the animals were initially tested on probe tests of increasing attentional demand by decreasing SDs (1.6, 1.0, 0.8, 0.6, 0.4, 0.2 s). Animals were then given tests of motor impulsivity by increasing delays (7, 9, 11, 13 s). Next, the animals were given tests of vigilance through increased session lengths (80 trials, followed by 140 trials). At each of these session lengths, the animals were tested on decreasing SDs (1.6, 1, 0.8, 0.6 s). In experiment 5, the test of 140 trials per session was repeated using 1.6, 1, 0.8, 0.6, 0.4, and 0.2 s SDs. Moreover, much recent focus has been on the therapeutic potentials of the partial α_7_ nicotinic acetylcholine receptor (α_7_nAChR) agonist EVP-6124 against the cognitive deficits of schizophrenia (Garay et al. 2016). In experiment 6, animals were treated with EVP-6124 (Latin-square design; 0, 3, 10, 30 mg/kg, 10 ml/kg, 30 min pre-treatment time, i.p; EnVivo Pharmaceuticals) and tested again on the 140 trials, 0.8-s SD, and 5-s delay parameter. Each dose-phase was separated by 48 h. Animals were tested drug-free on the baseline parameters (140 trials, 2-s SD, 5-s delay) on sessions separating each dose-phase. Prior to commencing the Latin-square, animals were given two saline injections for habituation to the injection procedure.

The dependent variables were completed trials, percent accuracy (total number of correct trials divided by total number of correct and incorrect trials), percent omission (total number of omitted trials divided by the total number of trials), percent premature responses (total number of premature trials divided by the total number of trials), percent perseverative correct (total number of perseverative responses to the correct location divided by the total number of correct responses), and average response latency and average reward retrieval latency. Each session was also divided into 10-trial bins, and the dependent measures were calculated within each 10-trial bin.

### Experiments 7–12: additional cognitive assays

#### Apparatus

The operant experiments used the same 32 touchscreen chambers that were used in experiments 4–6 (Campden Instruments, Loughborough, UK). The spontaneous object recognition experiment used two Y-shaped mazes made of Perspex.

#### Additional cognitive assays

Experiment 7 assessed discrimination learning and reversal learning in two separate two-choice challenges; the first using more discriminable stimuli and the second using more challenging stimuli (see Fig. 3a, b, inset). The task is described elsewhere (Mar et al. 2013). The dependent variables for reversal learning were trials to criterion, incorrect responses to criterion, correction trials to criterion, perseverative errors (the number of incorrect responses made before achieving >50 % correct responding in session), learning errors (the number of incorrect responses made after achieving ≤50 % correct responses correct responding in a session), average response latency, and average reward retrieval latency.

Experiment 8 assessed paired-associate learning (PAL). The task is extensively described elsewhere (Horner et al. 2013). Animals were tested for 70 sessions, and the data was collapsed into 5-session bins for analyses. The main dependent variables were percent accuracy (expressed as the number of correct responses divided by the total number of correct and incorrect responses), correction trials, average response latency, and average reward retrieval latency.

Experiment 9 assessed touchscreen progressive ratio (PR) by employing linear ramp schedules of 4, 8, 12, and 16 touches. The task is extensively described elsewhere (Heath et al. 2015). The dependent variables were the total number of trials completed, breakpoint (defined as the number of screen responses emitted in the last successfully completed trial of the session), the total number of screen touches emitted during the session, and the number of “blank” location touches recorded during the session (corrected for total session time).

Experiment 10 assessed touchscreen extinction learning. The task is extensively described elsewhere (Mar et al. 2013). Within each session, the dependent variables were percent responses (the total number of responses divided by the number responses and omissions), the number of touchscreen responses during the ITI, and stimulus response latencies.

Experiment 11 used the touchscreen trial-unique non-match to sample (TUNL) task to assess working memory through increasing delays (2, 4, 6, and 8 s) followed by tests of decreasing stimuli separations to assess pattern separation (small, medium, large). The task is extensively described elsewhere (Oomen et al. 2013; Kim et al. 2015). The dependent variables were percent accuracy (total number of correct trials dived by the total number of correct and incorrect trials), percent correction trials (total number of correction trials dived by the total number of correct and incorrect trials), average response latency, and average reward retrieval latency.

Experiment 12 assessed novel object recognition using 8 h (trial 1) and 11 h (trial 2 and trial 3) delays. The tasks are extensively described elsewhere (Winters et al. 2008). Video analyses of behaviour were made by an experimenter blind to the genotype and location of the novel object using JWatcher (version 1.0). For each animal, the discrimination ratio was calculated (time spent exploring the novel object divided by the total time spent exploring the objects). Total object exploration times in the sample-phase and object-biases were also calculated.

### Data analyses and statistics

Analyses were done using SPSS (v21.0, IBM Corp, Armonk, NY). Auditory-evoked neural responses were analysed using one-way between-subjects ANOVAs. Gabazine effect on pyramidal neuron firing was analysed with two-way ANOVA (with treatment and genotype as main factors). The behavioural data was analysed with repeated measured ANOVAs. Significant interactions were followed by one-way ANOVAs or Newman-Keuls post hoc comparisons.

## Results

### Experiment 1: baseline discharge rates

**Fig. 1:**
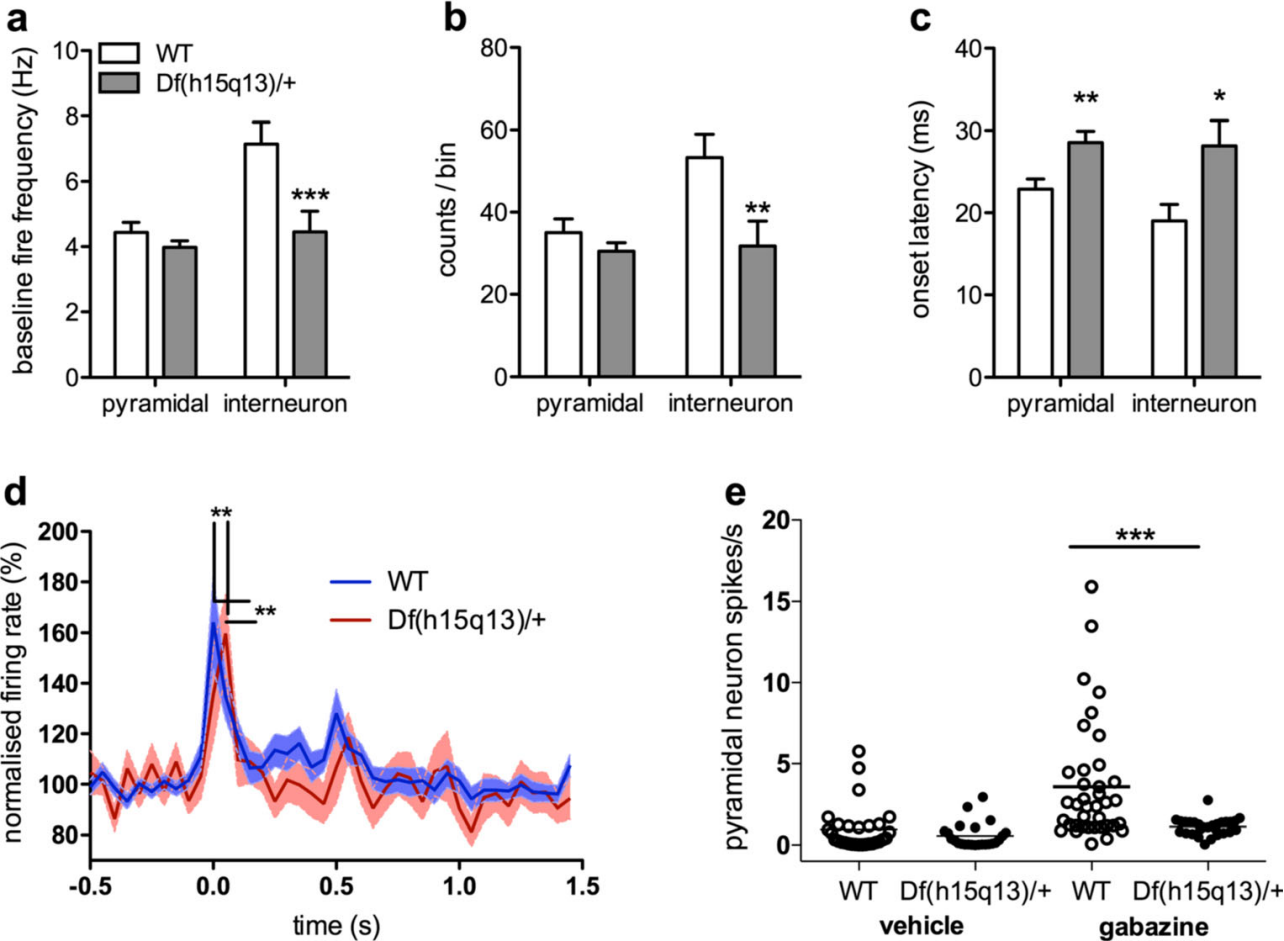
Prefrontal cortical electrophysiological characterisations of Df(h15q13)/+ and WT littermates. Data are presented as means ± SEM. **a** Baseline firing frequencies. The Df(h15q13)/+ mouse had attenuated baseline firing frequencies of PrL putative fast-spiking interneurons but not principal cells. **b** Response amplitudes. The Df(h15q13)/+ mouse had decreased detection amplitude in PrL putative fast-spiking interneurons but not principal cells. **c** Neuron onset latencies. The Df(h15q13)/+ had delayed onset latencies of both pyramidal cells and putative fast-spiking interneurons following auditory stimulation. **d** Average cortical putative fast-spiking interneuron response. Reduced firing rates and delayed onset of Df(h15q13)/+ prelimbic interneurons relative to WT mice in the auditory double-click paradigm. **e** Gabazine-induced elevation of PFC pyramidal spike frequency. The Df(h15q13)/+ showed decreased sensitivity to intra-mPFC GABA_A_R antagonism on pyramidal neuron spike frequency. *Asterisks* denote *p* < 0.05 (*p < 0.05, **p < 0.01, ***p < 0.001)

See Supplementary Figure 1a for waveform features used to classify units as putative pyramidal cells or putative fast-spiking interneurons. Df(h15q13/+) mice showed decreased baseline PrL interneuron discharge rate (Fig. 1a; *F*_1,94_ = 8.58, *p* = 0.004). There was no effect of genotype on baseline pyramidal neuron discharge rate (*F*_1,334_ = 1.71, *p* = 0.191).

### Experiment 2: cortical auditory-evoked neuron response

In the double-click paradigm (Fig. 1b–d), decreased neuron firing was observed in D(h15q13/+) interneurons (Fig. 1b; *F*_1,70_ = 6.74, *p* = 0.011) but not pyramidal neurons (*F*_1,239_ = 1.40, *p* = 0.238). Df(h15q13/+) interneurons (*F*_1,43_ = 6.64, *p* = 0.013) and pyramidal cells (*F*_1,146_ = 8.82, *p* = 0.003) also had increased onset latencies (Fig. 1c).

### Experiment 3: intra-mPFC GABA_A_R antagonism and pyramidal discharge rate

See Supplementary Figure 1b for average action potential durations of cells classified as pyramidal cells. The Df(h15q13/+) mutant showed decreased pyramidal neuron response to intra-mPFC GABA_A_R antagonism (Fig. 1e). A two-way ANOVA revealed significant main effects of gabazine (*F*_1,115_ = 14.63, *p* = 0.0002; Ns = 53 and 66 for saline and gabazine, respectively), and genotype (*F*_1,115_ = 12.02, *p* = 0.0007; Ns = 67 and 52 for WT and TG, respectively) and a significant gabazine × genotype interaction (*F*_1,115_ = 6.23, *p* = 0.020). Post hoc analysis (Newman-Keuls) revealed a significant difference in firing rates between gabazine and standard recording conditions in WT mice (0.97 ± 0.26 vs. 3.58 ± 0.59 spikes/s, *n* = 29 and 38, respectively; *p* < 0.00002) but not in Df(h15q13/+) mice (0.57 ± 0.16 vs. 1.12 ± 0.10 spikes/s; *n* = 24 and 28, respectively) (Fig. 1e). Hence, gabazine markedly elevated the discharge of putative pyramidal neurons in the PFC of WT mice, as previously observed in rats (Lladó-Pelfort et al. 2012) but not in Df(h15q13/+) mice. This suggests that the Df(h15q13/+) mouse has altered inhibitory neurotransmission in this cortical area.

### Experiments 4-6: the touchscreen 5-CSRTT

**Fig. 2:**
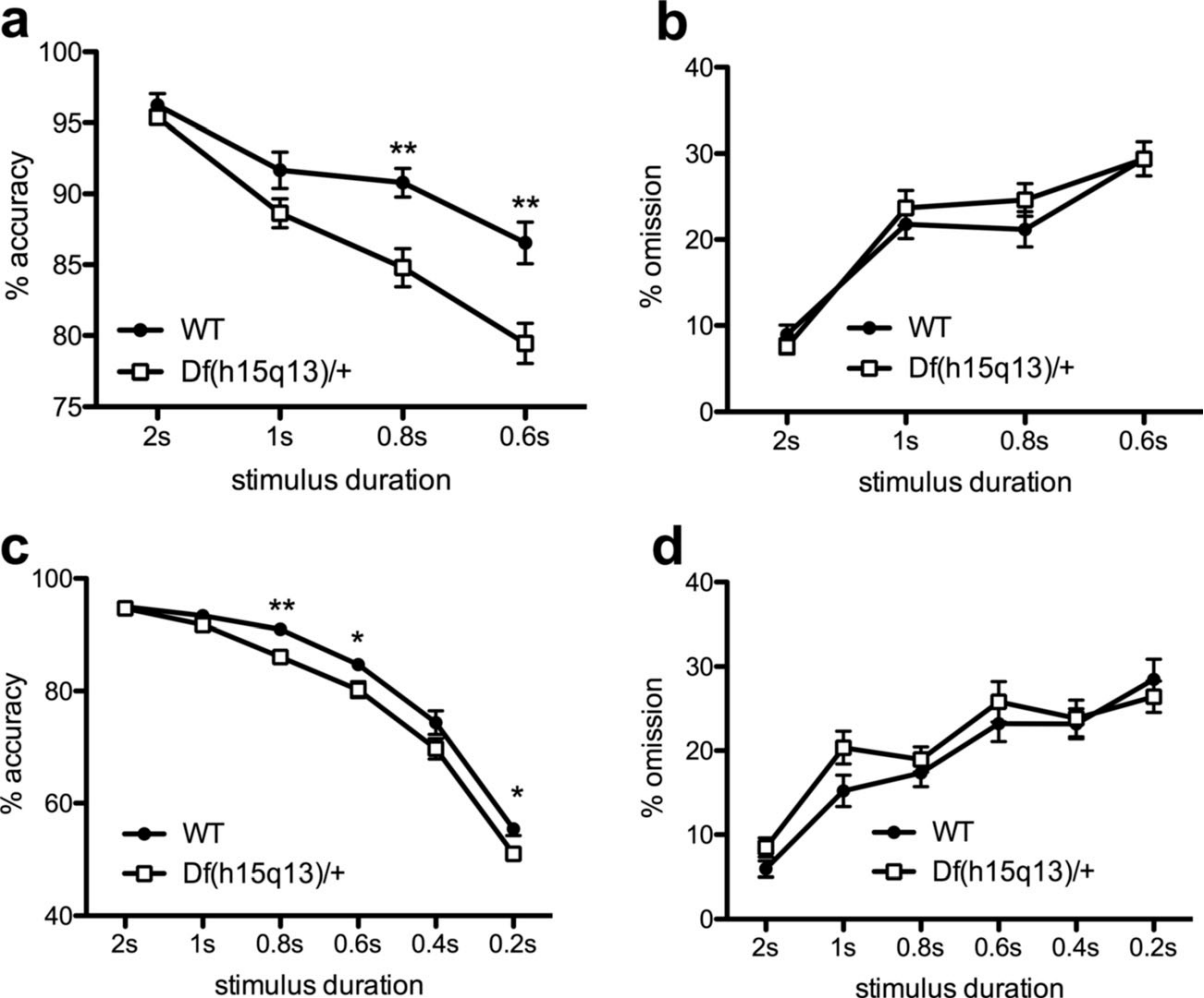
Performance of Df(h15q13)/+ and WT littermates in the 5-choice serial reaction time task. Data are presented as means ± SEM. **a**–**b** Test 1—decreasing stimulus durations. The Df(h15q13)/+ mouse showed lower accuracies at shorter stimulus durations (**a**) but did not differ from WTs on percent omission (**b**). **c**–**d** Test 2—decreasing stimulus durations. The accuracy impairment in the Df(h15q13)/+ mouse was reproduced using stimulus durations between 2 and 0.2 s (**c**). There was again no effect of genotype on percent omission (**d**). *Asterisks* denote *p* < 0.05 (*p < 0.05, **p < 0.01)

Df(h15q13/+) mice showed a selective stimulus duration-dependent accuracy deficit following extended training (Fig. 2a–d). When manipulating the stimulus duration, there were no effects of genotype or genotype by stimulus duration interactions on *initial* tests using 40- or 80-trial session lengths (Supplementary Fig. 2a–c). However, reproducible stimulus duration-dependent impairments were observed following extended training (∼100 sessions) using 140 trials per session. Extended training improved the performance of WT animals thereby producing increased room to detect the performance decrement of the Df(h15q13)/+ mice (see Supplementary Fig. 2a–c).

When tested on 140 trials per session, the Df(h15q13/+) mice showed decreased accuracies at shorter SDs (Fig. 2a, genotype: *F*_1,29_ = 11.08, *p* = 0.002, genotype × SD: *F*_3,87_ = 5.92, *p* = 0.001). The Df(h15q13/+) mouse showed lower accuracies at the 0.8-s (*p* = 0.001) and 0.6-s SDs (*p* = 0.002) but not at the 1-s (*p* = 0.074) and 2-s SDs (*p* = 0.375). There were no effects of genotype or genotype × SD interaction on percent omission (Fig. 2b) or any other behavioural measure (*p* ≥ 0.20; Supplementary Table S1). The accuracy impairment in the Df(h15q13/+) was reproduced in the same cohort of animals using stimulus durations between 2 and 0.2 s (Fig. 2c, d). Trial-bin analyses showed that Df(h15q13/+) mice had impaired accuracy throughout the length of the sessions. Thus, the deficit was not related to impaired vigilance (Supplementary Fig. 2a-c). No impairment was observed on test of increasing delays measuring impulsive responding (Supplementary Fig. 3d) and the partial α_7_nAChR agonist EVP-6124 did not reverse the accuracy impairment in the Df(h15q13/+) mouse (Table 1).

**Table 1:** Effect of systemic EVP-6124 on 5-CSRTT performance in WT and Df(h15q13)/+ mice

	% accuracy		% omission	
Dose (mg/kg)	WT	Df(h15q13)/+	WT	Df(h15q13)/+
0	90.45 ± 1.06	85.55 ± 1.46	13.24 ± 1.96	17.07 ± 1.90
1	90.14 ± 0.91	86.84 ± 1.25	13.77 ± 2.29	17.08 ± 1.96
10	90.06 ± 1.14	83.70 ± 2.02	14.95 ± 1.58	20.33 ± 3.36
30	87.38 ± 1.07	85.02 ± 1.41	20.88 ± 2.55	22.15 ± 2.77

Animals were tested using a 140-trial session length, 5-s delay, and 0.8-s SD. No effect of EVP-6124 on percent accuracy (genotype: *F*_1,30_ = 8.96, *p* = 0.004, dose: *F*_3,90_ = 2.12, *p* = 0.104, dose × genotype: *F*_3,90_ = 1.51, *p* = 0.216). EVP-6124 increased omissions at highest dose relative to vehicle (genotype: *F*_1,30_ = 1.97, *p* = 0.171, dose: *F*_3,90_ = 5.10, *p* = 0.003, dose × genotype: *F*_3,90_ = 0.43, *p* = 0.735) in both Df(h15q13)/+ and WT animals

### Experiments 7–12: additional cognitive

#### Experiment 7—visual discrimination and reversal learning

**Fig. 3:**
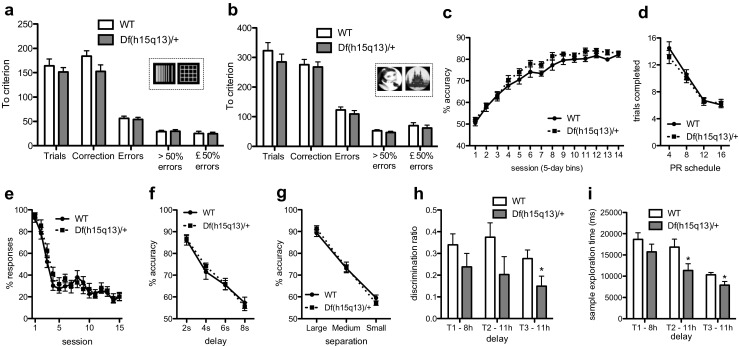
Performance of Df(h15q13)/+ and WT littermates on tests of cognitive flexibility, working memory, motivation and learning and memory. **a** Reversal learning test 1—“easy” discriminable stimuli. *Inset* depicts the stimuli. No effect of genotype on trials to criterion, correction trials to criterion, errors to criterion, early errors, or late errors (*p* ≥ 0.080). **b** Reversal learning test 2—“challenging” stimuli. No effect of genotype on trials to criterion, correction trials to criterion, errors to criterion, early errors, or late errors (*p* ≥ 0.277). **c** Paired-associate learning (PAL). No effect of genotype on percent accuracy (*p* ≥ 0.103). **d** Progressive ratio. No effect of genotype on trials completed (*p* ≥ 0.321). **e** Extinction learning. No effect of genotype on percent responses (*p* ≥ 0.385). **f** TUNL—delay challenge. No effect of genotype on percent accuracy (*p* ≥ 0.692). **g** TUNL—pattern separation challenge. No effect of genotype on percent accuracy (*p* ≥ 0.302). **h**–**i** Novel object recognition. The Df(h15q13/+) mouse showed reduced discrimination ratio at the 11-h delay (trial 2: *p* = 0.12, trial 3: *p* = 0.047) and reduced sample exploration time at the 11-h delay (trial 2: *p* = .036; trial 3: *p* = 0.023). *Asterisks* denote differences at which *p* < 0.05 (*p < 0.05)

There was no effect of genotype on visual discrimination learning (Supplementary Fig. S3a-b; *p* ≥ 0.441) or reversal learning (Fig. 3a, b; *p* ≥ 0.080).

#### Experiment 8—paired-associate learning (PAL)

There was no significant effect of genotype in the PAL task. There was no effect of genotype or genotype × session interaction on percent accuracy (Fig. 3c; *p* ≥ 0.103) or correction trials (Supplementary Fig. 3c; *p* ≥ 0.061).

#### Experiment 9—progressive ratio (PR)

Genotype did not affect measures of motivation in the PR task. There was no effect of genotype or genotype × PR schedule interaction on trials completed (Fig. 3d; *p* ≥ 0.321) or break points (Supplementary Fig. 3e; *p* ≥ 0.321).

#### Experiment 10—extinction learning

There were no effect of genotype or genotype × session interaction on percent responses in extinction learning (Fig 3e; *p* ≥ 0.385).

#### Experiment 11—trial-unique non-match to sample

When tested on increasing delays, there was no effect of genotype or genotype × delay interaction on percent accuracy (Fig. 3e; *p* ≥ 0.692). Similarly, there was no effect of genotype or genotype × separation interaction when tested using increasing stimuli separations (Fig. 3e; *p* ≥ 0.692). There were no effects of genotype on correction trials to criterion in either test (Supplementary Fig. 4f, g; *p* ≥ 0.683).

#### Experiment 12—novel object recognition (NOR)

The Df(h15q13/+) mouse showed impaired novel object recognition (Fig. 3h). However, this impairment was coupled with deceased sample exploration times (Fig. 3i) and can therefore not confidently be ascribed to a memory impairment. At the 8-h delay, there was no effect of genotype on sample exploration time or discrimination ratio (*p* ≥ 0.225). At the 11-h delay, the Df(h15q13/+) mouse showed decreased discrimination ratios (trial 2: *p* = 0.120; trial 3: *p* = 0.047) and decreased sample exploration times (trial 2: *p* = .036; trial 3: *p* = 0.023).

## Discussion

A chromosomal microdeletion at locus 15q13.3 is one of the largest known risk factors of schizophrenia and represents an ideal opportunity for back-translational study. We have previously generated a mouse model of the 15q13.3 microdeletion (Df(h15q13)/+) (Fejgin et al. 2014), and here, we characterised it using a variety of neurophysiological, pharmacological, and behavioural assays across multiple experimental sites. This comprehensive phenotyping revealed marked medial prefrontal cortical dysfunctions in the Df(h15q13)/+ model as measured by multiple parallel tests of inhibitory transmission and attentional functioning. These dysfunctions were observed as decreased baseline activity of putative interneuron and reduced interneuron and pyramidal neuron sensitivity to auditory stimulation. The Df(h15q13)/+ mouse also showed reduced sensitivity to intra-mPFC GABA_A_R blockade as measured by pyramidal neuron spike frequencies and a selective attentional impairment in the prefrontal cortical-dependent 5-CSRTT. Furthermore, we show that the attentional impairment of the Df(h15q13)/+ mouse is insensitive to systemic treatment with the partial α_7_nAChR agonist EVP-6124.

The Df(h15q13)/+ mouse showed reduced putative fast-spiking interneuron baseline activity and decreased putative pyramidal cell and fast-spiking interneuron auditory-evoked responses. These dysfunctions have clear translational relevance to schizophrenia and attentional dysfunction. For example, schizophrenia is associated with neurophysiological dysfunctions in passive sensory stimulation paradigms (2012) such as pre-attentative measures of mismatch negativity and repetition positivity (Baldeweg et al. 2004) and N100 amplitudes (Michie et al. 1990) as well as suppressed P50 gating (Adler et al. 1982), a phenomenon involving α_7_nAChRs (Freedman et al. 1997) coded by the *CHRNA7* gene for which the Df(h15q13)/+ mouse shows allelic insufficiency (Fejgin et al. 2014). Information processing deficits also include attenuated attentional allocation-driven cortical event-related potential P3b amplitudes (Roxborough et al. 1993; Weisbrod et al. 1999; Ford et al. 2001) and increased latencies of evoked potentials in visual and auditory paradigms, such as continuous performance tasks (Nieman et al. 2002) that reflect disrupted GABAergic (Benes and Berretta 2001) and cholinergic (Baldeweg et al. 2006) signalling efficacy. Reduction in cortical inhibition as measured by transcranial magnetic stimulation has also been observed in schizophrenic patients independent of clinical state (Fitzgerald et al. 2002; Daskalakis et al. 2002; Wobrock et al. 2008; Liu et al. 2009), and the phenotype is similarly linked to deficient cortical GABAergic transmission and attentional processes (Daskalakis et al. 2007). Analogous cortical neurophysiological dysfunction of auditory and visual event-related responses is also found in pharmacological animal models of schizophrenia (Bickel and Javitt 2009). Thus, our data indicate that the Df(h15q13)/+ mouse has decreased speed of processing and reduced recruitment of putative fast-spiking interneurons in the PrL and this phenotype corresponds with observations in schizophrenia patients. This also parallels our previous observations of reduced parietal, prelimbic, and hippocampal oscillatory activity or auditory-evoked potentials in the Df(h15q13)/+ mouse (Fejgin et al. 2014) which in conjunction with the current data would suggest more systemic speed of processing deficits in the mutant.

The Df(h15q13)/+ mouse showed prefrontal cortical GABA_A_R antagonist hyposensitivity with attenuated increase of putative pyramidal spike frequency in response to local (PrL + anterior cingulate) application of the GABA_A_R antagonist gabazine. The data parallel findings of disrupted GABAergic activity in many schizophrenia-relevant animal models, including cortical reductions in GABA-related markers (Morrow et al. 2007; Lodge et al. 2009; Francois et al. 2009; Ammassari-Teule et al. 2009) and aberrant pharmacological activation of fast-spiking interneurons (Tseng et al. 2008). The decreased sensitivity to PFC GABA_A_R blockade in Df(h15q13)/+ mice is also in agreement with histological findings in patients with schizophrenia showing reduced cortical expressions in a range of GABAergic markers, including non-pyramidal cells (1991), PV-immunoreactivity (Beasley and Reynolds 1997), GAD65/67 levels (Akbarian et al. 1995), and GAT1 (Volk et al. 2001). In sum, the attenuated putative pyramidal response to local GABA_A_R blockade in the Df(h15q13)/+ mice is in general agreement with data showing decreased GABAergic function in schizophrenia patients. The phenotype is also in agreement with the GABA_A_R antagonist hyposensitivity of Df(h15q13)/+ in seizure assays (Fejgin et al. 2014) and the decreased speed of processing and reduced baseline firing and decreased recruitment of putative fast-spiking interneurons in response to auditory stimulation.

We have seen no marked reduction of GABAergic markers that can account for the observed attenuation of putative interneuron basal activity or the decreased sensitivity of putative interneuron to auditory stimulation and pyramidal neuron sensitivity to GABA_A_R antagonism in the Df(h15q13)/+ mouse. In situ hybridization studies have nevertheless revealed a small but significant difference in prefrontal GAD expression in the Df(h15q13)/+ mutant (≤10 %; N. Santana, F. Artigas, unpublished observations). Notably, disruption of numerous transmitter processes affect cortical inhibitory control and pyramidal neuron sensitivity to GABAergic drugs (Haenschel and Linden 2001; Javitt et al. 2008; Kjaerby et al. 2014) and further work will be required to determine more fully the mechanisms underpinning the prefrontal cortical phenotype of the Df(h15q13)/+ mouse.

Importantly, the Df(h15q13)/+ mouse also exhibited a selective impairment in accuracy at shorter stimulus durations in the 5-CSRTT. This impairment was found after repeated testing improved the performance of WT animals which unmasked the deficit in the Df(h15q13)/+ mouse. The impairment was always observed throughout the sessions and therefore not related to reduced vigilance (see Supplementary Fig. S2a–c). Percent accuracy is considered a primary marker of attentional control in the 5-CSRTT. Percent omission and latency measures, which can reflect motivational, sensory, or motoric factors (Robbins 2002), were unaffected in the Df(h15q13)/+ mouse. The accuracy impairment was also only observed at shorter stimulus duration, further indicating that the phenotype is produced by impaired attention.

Attentional dysfunction is at the core of the cognitive deficits of schizophrenia (Marder and Fenton 2004; Carter and Barch 2007); it can precede other symptoms (Cornblatt et al. 1999), predict disorder development (2001), and be present in non-affected first-degree relatives (Snitz et al. 2006). Thus, the observation of similar attentional deficits in the Df(h15q13)/+ mouse suggests that the model carries translational utility within this cognitive domain with possible relevance for drug discovery.

An attentional impairment in the 5-CSRTT can also be predicted by the prelimbic neurophysiological and GABAergic phenotypes of the Df(h15q13)/+ mouse. In animals, intra-mPFC administration of GABA_A_R antagonists (Paine et al. 2011; Pehrson et al. 2013; Pezze et al. 2014) and agonists (Paine et al. 2011; Pezze et al. 2014) decrease percent accuracy in the 5-CSRTT, indicating that imbalances in cortical inhibitory activity disrupts attention. Moreover, in a variation of the 5-CSRTT, attenuated PrL inhibitory activity can predict accuracy while being unrelated to errors of omissions and premature responding (Totah et al. 2009). Our observations of attenuated PrL inhibitory activity together with a selective accuracy impairment in the Df(h15q13)/+ mice are entirely consistent with this view.

However, Df(h15q13)/+ and WT animals did not differ in performance on tasks dependent on cognitive flexibility, working memory, visuospatial learning, and motivation. The current behavioural experiments employed a battery approach where some animals where tested sequentially on multiple touchscreen tasks, and it may be that response strategies acquired in previous assays proactively interfered with performance on later tasks thereby masking more general effects of genotype on cognition. This is however unlikely.

First, the Df(h15q13)/+ mouse has previously been shown to exhibit a mild cognitive phenotype; the deletion fails to affect associative learning, spatial working memory, and sensorimotor gating (Fejgin et al. 2014). Visual discrimination and reversal learning, spatial working memory, and fear conditioning were also recently found to be unaffected in an alternative mouse model of the 15q13.3DS (D/+) (Kogan et al. 2015). Our observation of normal working memory, discrimination learning, and cognitive flexibility in the Df(h15q13)/+ mutant is in comprehensive agreement with these reports. Furthermore, reproducible attentional impairments were observed following extended testing in the 5-CSRTT. If extended testing masks some cognitive phenotypes in the Df(h15q13)/+ mutant the opposite pattern should be expected, deficits should be attenuated with repeated testing and not remain stable across multiple tests.

Moreover, although cortical GABAergic activity is relevant for a wide range of cognitive domains, mPFC-specific GABA_A_R antagonism through bicucilline fails to affect reversal learning (Enomoto et al. 2011) and working memory as assessed in the radial-arm maze (Enomoto et al. 2011) and T-maze (Herremans et al. 1996). Attentional function therefore appears particularly sensitive to GABAergic disruption (Enomoto et al. 2011; Tse et al. 2015). Nevertheless, under the present conditions, the lack of comprehensive impairment in these assays may limit the behavioural translational potential of the Df(h15q13)/+ mouse to attentional dysfunction.

Reduced α_7_nAChR expression has been advanced as a possible mechanism underlying the neuropsychiatric phenotypes of 15q13.3DS (LePichon et al. 2010; Hoppman-Chaney et al. 2013), and decreased cortical α_7_nAChR-expression is a mechanistic candidate for the Df(h15q13)/+ phenotype. α_7_nAChRs are expressed in parvalbumin-positive GABAergic fast-spiking interneurons (Murakami et al. 2013), *Chrna7* deletion causes dysregulated cortical inhibitory activity (Adams et al. 2012; Lin et al. 2014), and *Chrna7*^-/-^ mice show retarded performance in the 5-CSRTT (Young et al. 2004; Hoyle et al. 2006; Young et al. 2007).

However, acute dosing with the partial α_7_nAChR agonist EVP-6124 did not attenuate the attentional deficit in Df(h15q13)/+ mice. The lack of acute effect may be explained by reduced cortical α_7_nAChR expression leading to insufficient number of channels to be activated (full agonist treatment or chronic dosing may be required for efficacy) or by rapid agonist-induced receptor desensitisation leading to functional inactivation of the α_7_nAChR at the EVP-6124 doses used. Nevertheless, α_7_nAChR agonists have often failed to affect attentional measures in schizophrenic patients (Olincy et al. 2006; Freedman et al. 2008; Lieberman et al. 2013; Preskorn et al. 2014; Umbricht et al. 2014) and rodent 5-CSRTT performance (Grottick et al. 2003; Hahn et al. 2003; Hoyle et al. 2006). In accordance with this, we found EVP-6124 to be ineffective against the attentional impairment in the Df(h15q13)/+ mice. Thus, although decreased α_7_nAChR expression may cause cortical neurophysiological and attentional deficits, data indicate that α_7_nAChR agonism does not produce sufficient efficacy or that the mechanism underlying the phenotype may be downstream from the α_7_nAChR receptor or due to the functional importance of the deletion of other genes in the CNV region (Fejgin et al. 2014).

In sum, we observed multiple dysfunctions converging on disrupted medial prefrontal cortical functioning in the Df(h15q13)/+ mouse. These dysfunctions were observed as (i) decreased baseline firing and attenuated auditory-evoked responses of putative inhibitory interneurons and (ii) reduced putative pyramidal cell sensitivity to local GABA_A_R antagonism and (iii) selective decrease in percent accuracy in the 5-CSRTT. These data parallel the phenotypes of 15q13.3DS-related neuropsychiatric disorders and indicate that the Df(h15q13)/+ mouse has translational relevance for modelling some cortical dysfunctions associated with schizophrenia.

## Electronic supplementary material

Below is the link to the electronic supplementary material.

ESM 1 (DOCX 936 kb)
