# Supplementary material for: A mouse model of the 15q13.3 microdeletion syndrome shows prefrontal neurophysiological dysfunctions and attentional impairment
Source: Psychopharmacology (Berl). 2016 Mar 17;233:2151–63. doi: 10.1007/s00213-016-4265-2 (PMC4869740; doi:10.1007/s00213-016-4265-2)
Supplement: Supplementary file 1 — (DOCX 936 kb) [file 213_2016_4265_MOESM1_ESM.docx]

**SUPPLEMENTARY MATERIAL**

A mouse model of the 15q13.3 microdeletion syndrome shows prefrontal neurophysiological dysfunctions and attentional impairment

Simon RO. Nilsson^1,2,7^(PhD), Pau Celada^5,6^, Kim Fejgin^3^(PhD), Jonas Thelin^3,8^(PhD), Jacob Nielsen^3^(PhD), Noemí Santana^5,6^(PhD), Christopher J. Heath^1,2,4^(PhD), Peter H. Larsen^3^_­_(PhD), Vibeke Nielsen^3^(BSc), Brianne A. Kent^1,2^(PhD), Lisa M. Saksida^1,2^(PhD), Tine B. Stensbøl^3^(PhD), Trevor W. Robbins^1,2^(PhD), Jesper F. Bastlund^3^(PhD), Timothy J. Bussey^1,2*^(PhD), Francesc Artigas^5,6*^(PhD), Michael Didriksen^3*^(PhD).

^1^Department of Psychology, University of Cambridge, Cambridge, CB2 3EB, UK. ^2^MRC and Wellcome Trust Behavioural and Clinical Neuroscience Institute, University of Cambridge, Cambridge, CB2 3EB, UK. ^3^H. Lundbeck A/S, Synaptic Transmission, Neuroscience Research DK, Ottiliavej 9, Valby 2500, Denmark. ^4^Department of Life, Health and Chemical Sciences, The Open University, Walton Hall, Milton Keynes, MK7 6AA, UK. ^5^Institut d'Investigacions Biomèdiques de Barcelona, CSIC-IDIBAPS, Barcelona, Spain. ^6^Centro de Investigación Biomédica en Red de Salud Mental (CIBERSAM), Spain. ^7^Department of Psychology, State University of New York at Binghamton, Binghamton, NY 13902-6000, USA. ^8^Neuronano Research Center, Lund University, 223 81 Lund, Sweden. *These authors contributed equally to this work

*Corresponding author:* Department of Psychology, State University of New York at Binghamton, Binghamton, NY 13902-6000, USA. Email: sn440@cam.ac.uk. Tel: +1 (203) 444 6821

**Supplementary Figure 1a-b.** Identification of putative medial prefrontal cortical pyramidal neurons and interneurons. **(a) Identification based on action potential characteristics for analyses of baseline firing frequencies and auditory evoked neural responses.** The clustering of putative fast-spiking interneurons and putative pyramidal cells in awake mice was based on action potential waveform valley time-stamps using ‘valley to peak’ and ‘half valley width’. Putative fast-spiking interneurons were characterised by fast action potentials. **(b) Identification of putative pyramidal neurons based on action potential duration for analyses of response to GABA_A_ receptor antagonism.** The data is presented as mean ± SEM. Pyramidal neurons in control conditions showed similar action potential duration in WT and Df(h15q13)/+ mice. In presence of the gabazine leak, pyramidal neurons in WT mice showed significantly longer action potential duration compared with control conditions. * p<0.0003 vs. vehicle WT; #p<0.005 vs. gabazine WT. N = 29, 24, 38, and 28 neurons for WT, Df(h15q13)/+, gabazine WT and gabazine Df(h15q13)/+ groups, respectively.

**a**

**b**

**Supplementary Figure 2a-c.** The figure illustrates the unmasking of the attentional impairment in the Df(h15q13)/+ mouse in the 5-CSRTT through repeated testing using the 0.8s stimulus duration. The data is presented in 10-trial bins and represents mean accuracy ± SEM. Animals were initially tested on session lengths of 40 trials (a), followed by 80 trial (b), and 140 trials (c). With repeated testing, the performance of WT animals improved and this improvement was not matched by equal improvements in Df(h15q13)/+ animals. At this and other stimulus durations (data not shown), the Df(h15q13)/+ mouse was impaired throughout the sessions demonstrating that the attentional deficit was unrelated to impaired vigilance. Asterisk denote significant main effects of genotype at p < .05 (* p < .05, ** p < .01).

**a**

**c**

Test day ~54

Animal age: 19 weeks

Test day ~92

Animal age: 25 weeks

Test day ~105

Animal age: 27 weeks

**b**

**Supplementary Figure S3a-g.** Additional performance measures of WT and Df(h15q13)/+ littermates on behavioural assays. (a) Visual discrimination 1 using ‘easy’ discriminable stimuli. (b) Visual discrimination 2 using ‘challenging’ stimuli. **(c)** Number of correction trials over 70 sessions (presented in 5-day bins) in the PAL task. **(d)** Percent premature responses in tests of increasing delays in the 5-CSRTT. **(e)** Break points across four PR schedules. **(f-g)** Correction trials in the TUNL task when assessed over increasing delays (f) and decreasing separations (g). No significant effects of genotype (p ≥ 0.061) or genotype interaction with delay, session, schedule, or separation (p ≥ 0.283)

|  | | Genotype | |
| --- | --- | --- | --- |
| Stimulus  duration | Performance measure | WT | Df(15q13)/+ |
| 2s Trials completed | | 137.1 ± 1.67 | 139.2 ± 0.75 |
| % premature responses | | 2.16 ± 0.51 | 2.26± 0.45 |
| % perseverative correct | | 16.82 ± 2.10 | 16.01 ± 1.68 |
| Correct response lat. | | 1.08 ± .03 | 1.10 ± .03 |
| Reward retrieval lat. | | 1.23 ± .05 | 1.19 ± .04 |
|  | |  |  |
| 1s Trials completed | | 139.6 ± 0.30 | 139.5 ± 0.50 |
| % premature responses | | 1.18± 0.26 | 1.64 ± 0.40 |
| % perseverative correct | | 13.94 ± 1.86 | 13.76 ± 1.47 |
| Correct response lat. | | 0.90 ± .02 | 0.94 ± .02 |
| Reward retrieval lat. | | 1.15 ± .04 | 1.20 ± .05 |
|  | |  |  |
| 0.8s Trials completed | | 139.8 ± 0.19 | 139.0 ± 1.00 |
| % premature responses | | 1.77 ±0.65 | 2.32 ± 0.32 |
| % perseverative correct | | 15.81 ± 1.75 | 13.66 ± 1.32 |
| Correct response lat. | | 0.88 ± .02 | 0.88 ± .02 |
| Reward retrieval lat. | | 1.16 ± .06 | 1.20 ± .04 |
|  | |  |  |
| 0.6s Trials completed | | 134.1 ± 3.07 | 139.6 ± 0.37 |
| % premature responses | | 1.85 ± 0.28 | 3.07 ± 0.49 |
| % perseverative correct | | 16.77 ± 2.51 | 14.58 ± 1.57 |
| Correct response lat. | | 0.81 ± .02 | 0.82 ± .02 |
| Reward retrieval lat. | | 1.16 ± .05 | 1.20 ± .06 |

No significant effects of genotype (p ≥ 0.202) or genotype × stimulus duration interactions (p ≥ 0.561).

Supplementary Table S1. Secondary performance measures of WT and Df(15q13)/+ littermates in tests of decreasing stimulus durations in the 5-CSRTT (Experiment 4).
